# Selective Inhibition of Janus Kinase 3 Has No Impact on Infarct Size or Neurobehavioral Outcomes in Permanent Ischemic Stroke in Mice

**DOI:** 10.3389/fneur.2017.00363

**Published:** 2017-07-25

**Authors:** Kelly M. DeMars, Sean C. Pacheco, Changjun Yang, David M. Siwarski, Eduardo Candelario-Jalil

**Affiliations:** ^1^Department of Neuroscience, McKnight Brain Institute, University of Florida, Gainesville, FL, United States

**Keywords:** Janus kinase 3, ischemic stroke, decernotinib (VX-509), middle cerebral artery occlusion, neuroinflammation

## Abstract

Janus kinase 3 (JAK3) is associated with the common gamma chain of several interleukin (IL) receptors essential to inflammatory signaling. To study the potential role of JAK3 in stroke-induced neuroinflammation, we subjected mice to permanent middle cerebral artery occlusion and investigated the effects of JAK3 inhibition with decernotinib (VX-509) on infarct size, behavior, and levels of several inflammatory mediators. Results from our double immunofluorescence staining showed JAK3 expression on neurons, endothelial cells, and microglia/macrophages in the ischemic mouse brain (*n* = 3). We found for the first time that total and phosphorylated/activated JAK3 are dramatically increased after stroke in the ipsilateral hemisphere (**P < 0.01; *n* = 5–13/group) in addition to increased *IL-21* expression after stroke (**P < 0.01; *n* = 5–7/group). However, inhibition of JAK3 confirmed by reduced phosphorylation of its activation loop at tyrosine residues 980/981 does not reduce infarct volume measured at 48 h after stroke (*n* = 6–10/group) nor does it alter behavioral outcomes sensitive to neurological deficits or stroke-induced neuroinflammatory response (*n* = 9–10/group). These results do not support a detrimental role for JAK3 in acute neuroinflammation following permanent focal cerebral ischemia. The functional role of increased JAK3 activation after stroke remains to be further investigated.

## Introduction

An ischemic stroke is caused by a clot in the cerebrovasculature that starves downstream tissue of oxygen and nutrients. The infarct core is the tissue immediately downstream of the blockage in which cell death occurs within minutes, and the penumbra is the tissue surrounding the core that dies as a result of spreading neuroinflammation. Resident microglia become activated and release cytokines and chemokines, and immune cells such as monocyte-derived macrophages, leukocytes, and neutrophils are recruited to the site. These immune cells exacerbate the extent of injury, particularly by secreting matrix metalloproteinases that degrade the blood–brain barrier and can thus encourage hemorrhagic transformation and increase free radical formation in the brain parenchyma.

Janus kinase 3 (JAK3), a tyrosine kinase activated by interleukin (IL) receptors that use the common gamma chain (IL-2, IL-4, IL-7, IL-9, IL-15, and IL-21) (1), is predominately expressed on hematopoietic cells to modulate cell signaling in response to cytokines and is therefore vital to initiating an immune response. When one of the aforementioned cytokines binds to its receptor, the induced conformational change leads to auto- or transphosphorylation of the associated JAK3 activation loop at Y980/981 (2). JAK3 can then go on to phosphorylate various signal transducers and activators of transcription (STATs) that dimerize and translocate into the nucleus to induce inflammatory gene transcription (3).

Janus kinase 3 knockout mice are immunocompromised due to their non-functional T- and B-cells. It has been shown that JAK3 mRNA is upregulated in ischemic stroke (4), and JAK3 signaling facilitates IL-8-mediated neutrophil chemotaxis (5). Furthermore, the selective JAK3 inhibitor, JANEX-1, reduced infarct size and reperfusion injury in a mouse model of myocardial ischemia (6). Because RAG1^−/−^, CD4+ T-cell^−/−^, and CD8+ T-cell^−/−^ mice have reduced infarcts compared to wild-type controls subjected to transient middle cerebral artery occlusion (MCAO) (7), inhibiting JAK3 pharmacologically may have a similar neuroprotective effect. Administration of tofacitinib, a JAK1/JAK3 inhibitor, reduced neurological deficits in mice subjected to transient focal cerebral ischemia (8).

There has been growing interest in developing highly selective JAK3 inhibitors to combat inflammation with minimal side effects (9, 10). JAK3 inhibition has been shown to be efficacious at ameliorating inflammation in a multitude of diseases (10–12). Decernotinib (VX-509) inhibits JAK3-mediated signaling both *in vitro* and *in vivo* with a high potency and selectivity and is clinically used to treat rheumatoid arthritis (9, 10).

In this study, we hypothesized that JAK3 inhibition with decernotinib would reduce infarct size and improve neurological function in a mouse model of ischemic stroke. Of the six ILs that use the common gamma chain, we identified increased *IL-21* expression in the ipsilateral cortex in addition to confirming that our model of ischemic stroke induces significant neuroinflammation with qRT-PCR for several inflammatory mediators. We are the first to show that total and phosphorylated JAK3 (pJAK3)/activated JAK3 levels are dramatically increased in response to ischemic stroke. Although it is able to reduce the stroke-induced increase in pJAK3 and STAT3, downstream effectors of JAK3 activation, decernotinib is not neuroprotective in the acute phase of ischemic stroke. Overall, our study could not identify JAK3 as a relevant therapeutic target to ameliorate neuroinflammation and reduce brain injury in permanent ischemic stroke.

## Materials and Methods

### Animals and Experimental Ischemic Stroke Surgery

All animal procedures were performed in accordance with approved guidelines of the National Institutes of Health for the Care and Use of Laboratory Animals, the ARRIVE guidelines (https://www.nc3rs.org.uk/arrive-guidelines), and the guidelines approved by the Institutional Animal Care and Use Committee at the University of Florida (protocol #201607934). Adult male mice (between the ages of 9 and 12 weeks; weighing between 25 and 30 g; C57BL/6N from Taconic Biosciences, Hudson, NY, USA) were housed within the university’s animal facility on a 12 h light/dark cycle with free access to food and water. Animals were acclimated to the facility for at least 1 week before experiments.

Mice that were randomly assigned to treatment groups using the Graphpad randomization tool (http://www.graphpad.com/quickcalcs/randomize1.cfm) were anesthetized in 2–2.5% isoflurane in medical grade oxygen for surgical procedures. Tandem permanent ligation of the left middle cerebral artery (MCA) and common carotid artery was performed as detailed in a previous report (13). Interruption of blood flow was visually confirmed in each animal. Mice were given 0.05 mg/kg buprenorphine hydrochloride subcutaneously for an analgesic and placed in a temperature-controlled chamber to recover. This surgical technique consistently yields an infarct limited to the cortical tissue with 0% mortality rate. For sham surgery, all procedures were kept constant except for MCA ligation.

### Drug Treatment

Animals were treated with vehicle or 0.03, 0.1, 0.3, 1.0, 3.0, or 10 mg/kg decernotinib (Cat # CT-DECE; ChemieTek, Indianapolis, IN, USA), a highly selective JAK3 inhibitor. This dose range was selected based on previous literature inhibiting JAK3 in models of peripheral inflammation (10). Based on an *a priori* analysis using G*Power 3.1 (14), we needed a minimum of eight subjects per group to achieve 90% power for infarct calculation. A stock solution of decernotinib was prepared in dimethyl sulfoxide (DMSO), diluted to appropriate doses using physiological saline, and injected intraperitoneally immediately after MCA ligation, 18 h postischemia, and finally 24 h postischemia for infarct calculation experiments. Mice in the vehicle group instead received an equivalent amount of DMSO dissolved in physiological saline on the same drug schedule. All treatments were given by an investigator blinded to experimental groups (coded animals and drug vials). To confirm decernotinib was inhibiting JAK3 and STAT3 activation in the brain, we performed permanent middle cerebral artery occlusion (pMCAO), immediately after injected vehicle or 10 mg/kg decernotinib intraperitoneally, injected again at 18 h, and sacrificed the animals 24 h post-MCAO for immunoblotting analyses.

### Brain Tissue Collection and Infarct Size Calculation

Mice were sacrificed 24 h postischemia for Western blotting, qRT-PCR, and double immunofluorescence or 48 h postischemia for infarct calculation. Mice were deeply anesthetized with intraperitoneal injection of 150 mg/kg pentobarbital and intracardially perfused with either ice-cold 0.9% saline for Western blotting experiments, saline containing 1% procaine hydrochloride for the infarct calculation study, or saline containing 1% procaine hydrochloride followed by Histochoice^®^ (Cat # H112; Amresco, LLC, Solon, OH, USA) to fix the tissue for immunohistochemistry. For double immunofluorescence, the brain was removed and placed in Histochoice^®^ for 24 h, equilibrated in 30% sucrose in phosphate-buffered saline (PBS), frozen in Tissue-Tek^®^ O.C.T. compound (Cat # 25608-930; VWR, Radnor, PA, USA), and kept at −20°C until cryosectioned at 20 µm. For infarct calculation, brains were removed and placed in a mouse brain matrix and sliced into six 1 mm sections which were then incubated in 2% 2,3,5-triphenyl-tetrazolium chloride (TTC) in PBS for 30 min at room temperature to delineate live and dead brain tissue. Infarct volume was calculated as detailed in our previous reports (15, 16) by an investigator blinded to treatment groups (coded images). For immunoblotting and qRT-PCR experiments, a 4-mm thick coronal slice, which included the center of the stroke lesion, was dissected into ipsilateral and contralateral hemispheres, and the cerebral cortex was removed, immediately placed in RNAlater^®^ (Cat # R0901; Sigma- Aldrich, St. Louis, MO, USA) and stored at −80°C.

### Tissue Homogenization and Western Blotting

Brain tissue processing for immunoblotting was performed as detailed in our previous publications (15, 16). Fifty microgram total protein of brain homogenate in Laemmli buffer containing 2.5% β-mercaptoethanol per lane was incubated at 70°C for 5 min for STAT3 and phosphorylated STAT3 immunoblots and separated on a 4–20% gradient Mini-PROTEAN TGX™ gel (Cat # 456-1096; BioRad, Hercules, CA, USA) for 45 min at 200 V and transferred onto nitrocellulose membranes using the Trans-Blot^®^ Turbo™ Transfer System (BioRad) for 30 min at 25 V. Membranes were washed once with Tris-buffered saline containing 0.1% Tween^®^-20 (TBST), blocked for 1 h at room temperature in Odyssey blocking buffer (Cat # 927-50000; Li-Cor, Lincoln, NE, USA) for pJAK3, total JAK3, total STAT3, and phosphorylated STAT3 or 5% non-fat milk in Tris-buffered saline (TBS) containing 0.05% Kathon™ for β-actin. Membranes were washed once more with TBST and probed with 1:1,000 mouse anti-JAK3 (Cat # MA5-15561; ThermoFisher Scientific, Waltham, MA, USA), 1:500 rabbit anti-phosphoJAK3 (Cat # 5031; Cell Signaling Technology, Danvers, MA, USA), 1:1,000 mouse anti-STAT3 (Cat # 9139; Cell Signaling Technology), 1:1,000 rabbit anti-phosphoSTAT3 (Cat # 9145; Cell Signaling Technology) in Odyssey blocking buffer, and 1:10,000 mouse anti-β-actin (Cat # A1978; Sigma-Aldrich) in 5% milk in TBST overnight at 4°C. The next day, membranes were washed four times with TBST for 5 min each and probed for 1 h at room temperature with 1:30,000 goat anti-mouse IRDye 800CW (Cat # 925-32210; Li-Cor) for JAK3 detection and 1:40,000 donkey anti-mouse IRDye 680RD (Cat # 925-68072; Li-Cor) for β-actin detection. Finally, membranes were washed four times with TBST for 5 min and placed in TBS. Membranes were scanned using the Odyssey Imager (Cat # 9120; Li-Cor) and band intensity was quantified with ImageJ (NIH, Bethesda, MD, USA).

### RNA Isolation and qRT-PCR

Total RNA was isolated using a modified version of the Chomczynski and Sacchi method of guanidine thiocyanate-phenol-chloroform RNA extraction (17). About 80 mg of tissue was removed from RNAlater and placed in 600 µL radioimmunoprecipitation buffer containing protease and phosphatase inhibitor and 0.5 M EDTA at 10 µL/mL lysis buffer and homogenized with a Tissue Tearor (Cat # 985370; BioSpec, Inc., Bartlesville, OK, USA). Next, 300 µL guanidine thiocyanate solution containing 0.7% β-mercaptoethanol was added to 300 µL of the homogenate. Sixty microliters of 2 M NaOAc pH 4.0 and 600 µL of water-saturated phenol were added to the homogenate, thoroughly vortexed, and incubated at room temperature for 5 min before spinning down at 10,000 × *g* for 20 min at 4°C. The aqueous phase was then precipitated with an equal volume of isopropanol at −20°C for 30 min. The samples were centrifuged again at 10,000 × *g* for 15 min at 4°C, the supernatant was removed, and the pellet was dissolved in 75 µL nuclease-free water in a heating block at 50°C for 5 min. For DNase digestion, 13 µL of nuclease-free water, and 2 µL of DNase I (Cat # 6344; 2,220 μg/mL; Worthington Biochemical Corporation, Lakewood, NJ, USA) were added to 10 µL of 10× DNAse I Reaction Buffer (Cat # B0303S; New England BioLabs^®^ Inc., Ipswich, MA, USA) to each sample and incubated at 37°C for 1 h. Next, 33 µL 8 M LiCl was added to the sample and precipitated for 30 min at −20°C. The solution was next centrifuged at 16,000 × *g* for 20 min at 4°C, the supernatant was decanted, and the pellet was washed with 200 µL 70% ethanol precooled at −20°C and centrifuged once more at 16,000 × *g* for 10 min at 4°C. Finally, the supernatant was decanted, the pellet was allowed to air-dry for 5 min at room temperature, and dissolved in 30 µL of nuclease-free water in a heating block set to 50°C for 1 min. The samples were thoroughly vortexed and placed on ice. Concentration and purity were measured on a Take3 Micro-Volume Plate (BioTek Instruments, Winooski, VT, USA) using Gen5 Data Analysis version 2.00.18 (BioTek Instruments). Total RNA of 1 μg was reverse transcribed using iScript™ Reverse Transcription Supermix (Cat # 1708841; BioRad) and diluted to 10 ng/µL with IDTE buffer pH 8.0 (Cat # 11-05-01-13; Integrated DNA Technologies, Coralville, IA, USA). Twenty or forty nanogram cDNA was combined with exon-exon spanning forward and reverse primers at 500 µM (Integrated DNA Technologies) per reaction and either PerfeCTa^®^ SYBR^®^ Green Fastmix^®^ (Cat # 95072-012; Quanta Biosciences, Beverly, MA, USA) or Luna Universal qPCR Master Mix (Cat # M3003L; New England BioLabs^®^ Inc.). Reactions were run in triplicate on a BioRad CFX96 Touch Real-Time PCR Detection System. For PerfeCTa reactions, the polymerase activation/DNA denaturation phase was performed at 95°C for 30 s, before 40 cycles of denaturing at 95°C for 5 s and annealing at 60°C for 30 s. For Luna Reactions, the polymerase activation/DNA denaturation phase was performed at 95°C for 1 min, then 40 cycles of denaturing at 95°C for 15 s and annealing at 60°C for 30 s. Cycle threshold (Ct) values were normalized to peptidylprolyl isomerase A (*Ppia*) because Ct values of this housekeeping gene do not significantly vary between treatment groups (data not shown). The primer sequences are described in Table 1. Gene expression is reported as a fold change normalized to the contralateral vehicle group.

**Table 1:** Name and sequence of primers used for quantitative real-time PCR.

Gene	Accession Number	Forward	Reverse
*IL-2*	NM_008366	5′-GCAGGATGGAGAATTACAGGAA-3′	5′-GCAGAGGTCCAAGTTCATCTTC-3′
*IL-4*	NM_021283	5′-GAACGAGGTCACAGGAGAAG-3′	5′-ACCTTGGAAGCCCTACAGA-3′
*IL-7*	NM_008371	5′-CCGCAGACCATGTTCCAT-3′	5′-GTCTTTAATGTGGCACTCAGATG-3′
*IL-9*	NM_008373	5′-CCAATGCCACACAGAAATCAAG-3′	5′-TGGTCTGGTTGCATGGC-3′
*IL-15*	NM_001254747	5′-TCTCGTGCTACTTGTGTTTCC-3′	5′-CATCTATCCAGTTGGCCTCTG-3′
*IL-21*	NM_021782	5′-TGACTTGGATCCTGAACTTCTATC-3′	5′-GGTTTGATGGCTTGAGTTTGG-3′
*Ccl2*	NM_011333	5′-CATCCACGTGTTGGCTCA-3′	5′-AACTACAGCTTCTTTGGGACA-3′
*Cxcl1*	NM_008176	5′-CCAAACCGAAGTCATAGCCA-3′	5′-GTGCCATCAGAGCAGTCT-3′
*Cxcl10*	NM_021274	5′-ATTTTCTGCCTCATCCTGCT-3′	5′-TGATTTCAAGCTTCCCTATGGC-3′
*IL-1β*	NM_008361	5′-GACCTGTTCTTTGAAGTTGACG-3′	5′-CTCTTGTTGATGTGCTGCTG-3′
*Ptgs2*	NM_011198	5′-CAAGACAGATCATAAGCGAGGA-3′	5′-GCGCAGTTTATGTTGTCTGTC-3′
*Mmp-9*	NM_013599	5′-GACATAGACGGCATCCAGTATC-3′	5′-GTGGGAGGTATAGTGGGACA-3′
*Tnfα*	NM_013693	5′-AGACCCTCACACTCAGATCA-3′	5′-TCTTTGAGATCCATGCCGTTG-3′
*IL-6*	NM_031168	5′-AGCCAGAGTCCTTCAGAGA-3′	5′-TCCTTAGCCACTCCTTCTGT-3′
*Ppia*	NM_008907.1	5′-GGCCGATGACGAGCCC-3′	5′-TGTCTTTGGAACTTTGTCTGCAA-3′

*IL, interleukin; Ccl2, chemokine (C-C motif) ligand 2; Cxcl1, chemokine (C-X-C motif) ligand 1; Cxcl10, chemokine (C-X-C motif) ligand 10; Ptgs2, prostaglandin-endoperoxidase synthase 2; Mmp-9, matrix metalloproteinase-9; Tnfα, tumor necrosis factor α; Ppia, peptidylprolyl isomerase A*.

### Double Immunofluorescence

Brains were cryosectioned at 20 µm and permeabilized with two washes of PBS with 0.1% Triton™ X-100 (PBST) for 5 min, and blocked for 1 h at room temperature with PBST containing 1% bovine serum albumin and 5% normal goat serum. Slides were incubated overnight at 4°C with mouse anti-JAK3 (1:100, Cat # MA5-15561; ThermoFisher) and one of the following established cell markers: rabbit anti-Iba1 (1:100, Cat 019-19741; Wako Chemicals USA, Inc., Richmond, VA, USA) to identify microglia/macrophages, rabbit anti-GFAP (1:100, Cat # Z0334; Dako North America, Inc., Carpinteria, CA) to identify astrocytes, rabbit anti-NeuN (1:100, Cat # NBP1-77686SS; Novus Biologicals, LLC, Littleton, CO, USA) to identify neurons, or rat anti-CD31 (1:100; Cat # NB600-1475; Novus Biologicals) to identify endothelial cells. Slides were then washed three times with PBST for 5 min and blocked again for 20 min at room temperature. Next, sections were incubated with goat anti-mouse Alexa Fluor 594 (1:250, Cat # A11032; ThermoFisher) and either goat anti-rat Alexa Fluor 488 (1:250, Cat # A11006; ThermoFisher) or goat anti-rabbit Alexa Fluor 488 (1:250, Cat # 111-545-144; Jackson ImmunoResearch Laboratories, West Grove, PA, USA) for 90 min at room temperature. Finally, tissue was washed three times with PBST for 5 min, counterstained with 100 nM DAPI in PBS for 10 s, rinsed three times with water, and coverslipped with Fluoromount™ (Cat # F4680; Sigma-Aldrich). Images were acquired with a spinning disk confocal microscope (Cat # DSU-IX81; Olympus, Center Valley, PA, USA) at 60×. Three ischemic animals were used for immunohistochemical analyses to identify cells expressing JAK3 after stroke.

### Adhesive Removal Test

To measure sensorimotor function before (baseline performance) and at 24 h after stroke, we performed the adhesive removal test (18).

### Statistical Analysis

All data were analyzed with GraphPad Prism 5 and depicted as mean ± SEM. A two-way ANOVA with a Bonferroni post-test or an unpaired t-test were used to analyze Western blot, qRT-PCR, and behavioral results. A one-way ANOVA with a Dunnett’s Multiple Comparison post-test was used to analyze infarct volume. A *P* value <0.05 was regarded as statistically significant.

## Results

**Figure 1:**
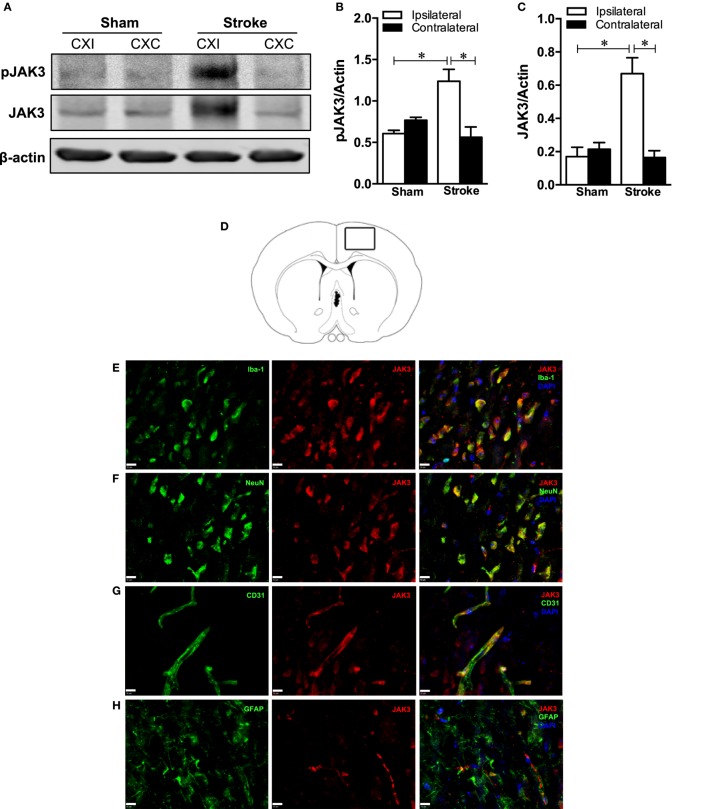
**(A)** Representative Western blots depicting phosphorylated JAK3 (pJAK3), total JAK3, and corresponding β-actin levels in the ipsilateral (CXI) versus the contralateral (CXC) cortex at 24 h in sham and mice subjected to permanent middle cerebral artery occlusion (pMCAO). **(B,C)** Quantified pJAK3 and JAK3 values normalized to β-actin in the cortex of sham and mice subjected to stroke. Both pJAK3 and JAK3 are significantly increased in the ipsilateral cortex of stroked animals (*P < 0.05 and **P < 0.01 with respect to sham ipsilateral/contralateral and stroke contralateral; sham, *n* = 5; stroke, *n* = 13). Data were analyzed using a two-way ANOVA (ipsilateral/contralateral or sham/stroke) with Bonferroni post-test. **(D)** Diagram showing peri-infarct brain region from which images were taken (*n* = 3). Scale bar is 10 µm, and magnification is 60×. **(E)** JAK3 colocalized with microglia/macrophages labeled with Iba-1. **(F)** JAK3 colocalized with neurons labeled with NeuN. **(G)** JAK3 colocalized with endothelial cells labeled with CD31. **(H)** JAK3 is not colocalized with astrocytes labeled with GFAP.

We first investigated the effects of ischemic stroke on total and pJAK3/activated JAK3 levels in the cerebral cortex of mice subjected to pMCAO using immunoblotting. We found that both pJAK3 at tyrosine residues 980/981 and total JAK3 were dramatically increased in the ipsilateral cortex compared to sham and contralateral levels in brain tissue 24 h poststroke (Figures 1A–C). Next, we localized JAK3 expression to macrophages/microglia (Iba1+), neurons (NeuN+), and endothelial cells (CD31+) in peri-infarct regions of the ipsilateral cortex 24 h postischemia to elucidate which brain cells would be affected by decernotinib treatment (Figures 1D–G). We could not find JAK3 expression on astrocytes with our methods (Figure 1H).

**Figure 2:**
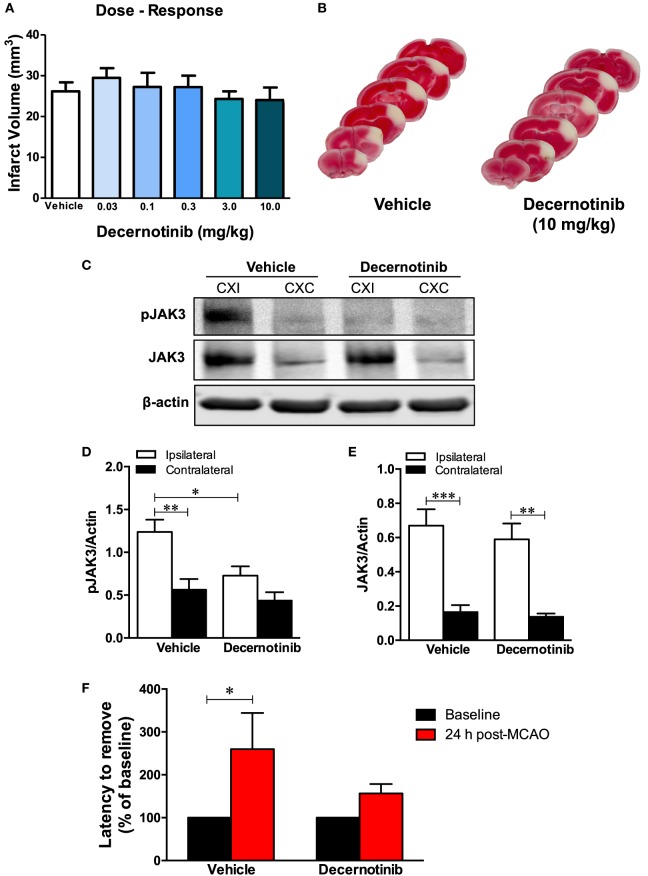
**(A)** Dose–response curves measuring infarct volume in vehicle-treated and 0.03, 0.1, 0.3, 3, and 10 mg/kg of decernotinib-treated mice. There was no significant difference in infarct size across groups (vehicle, *n* = 8; 0.03 mg/kg, *n* = 6; 0.1 mg/kg, *n* = 8; 0.3 mg/kg, *n* = 8; 3 mg/kg, *n* = 10; and 10 mg/kg, *n* = 8). Data were analyzed using a one-way ANOVA with a Dunnett’s multiple comparison post-test. **(B)** Representative 2,3,5-triphenyl-tetrazolium chloride (TTC) images of vehicle- and 10 mg/kg decernotinib-treated mice to show extent of cortical injury in our model of permanent middle cerebral artery occlusion (MCAO). **(C)** Representative immunoblots of brain tissue obtained from the ipsilateral (CXI) and contralateral (CXC) cerebral cortex depicting reduced phosphorylation of Janus kinase 3 (JAK3) in the 10 mg/kg decernotinib group compared to vehicle. There is no effect of decernotinib on stroke-induced increase in total JAK3 levels. **(D,E)** Graphical summary of immunoblot data (*P < 0.05 compared to vehicle ipsilateral; vehicle, *n* = 9; 10 mg/kg decernotinib, *n* = 5). Data were analyzed using a two-way ANOVA. **(F)** Latency to remove the sticky tape from the contralateral paw, measuring sensorimotor deficits, in vehicle and decernotinib groups. Stroke induced a significant increase in the latency to remove the sticker at 24 h in the vehicle-treated group. Comparing both treatments at 24 h, there was a trend toward a better performance in the adhesive removal test in mice given decernotinib, but this effect did not reach a statistical significance (two-way ANOVA with Bonferroni post-test; vehicle *n* = 9; decernotinib at a dose of 10 mg/kg, *n* = 10).

Finally, we performed a detailed dose–response analysis measuring infarct size with differing doses of decernotinib, and found that inhibiting JAK3 is not neuroprotective in the acute phase of experimental ischemic stroke in the mouse (Figures 2A,B) since it had no significant effect on infarct size at 48 h regardless of the dose from 0.03 to 10 mg/kg.

Since we administered decernotinib intraperitoneally, it was important to confirm target engagement and that the drug reached the brain to inhibit JAK3 activation in the ischemic area. We established decernotinib was inhibiting JAK3 in the brain by performing immunoblots that measured a dramatic reduction in phosphorylation of JAK3’s activation loop at tyrosine residues Y980/981 with 10 mg/kg of decernotinib compared to vehicle-treated mice subjected to pMCAO (Figures 2C,D). Decernotinib did not alter stroke-induced total JAK3 levels (Figure 2E).

Additionally, although we show JAK3 inhibition in the brain with 10 mg/kg decernotinib, behavioral analysis indicates that treatment does not yield neurological protection since there was no significant difference between vehicle- and decernotinib-treated groups in the latency to remove the sticker in the adhesive removal test after pMCAO (Figure 2F).

**Figure 3:**
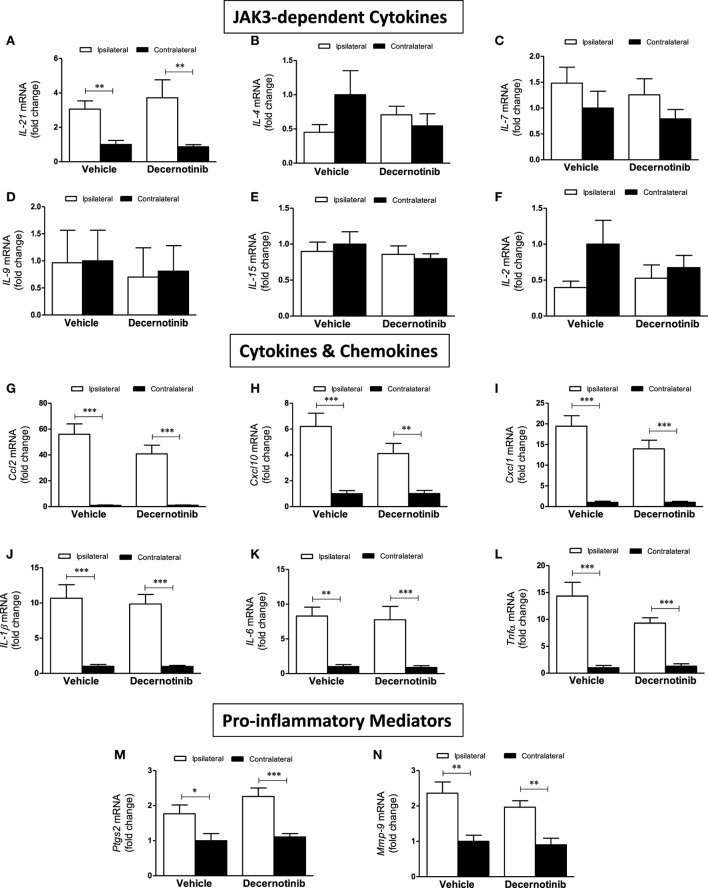
**(A)** *IL-21* is increased in the ipsilateral cortex compared to the contralateral cortex (**P < 0.01) **(B)** *IL-4* does not appreciably change between treatment groups or hemisphere. **(C)** There is a trend toward increased *IL-7* in the ipsilateral cortex. **(D)** There is no change in *IL-9* between groups. **(E)** There is no change in *IL-15* between groups. **(F)** There is no significant change in *IL-2* across groups. **(G)** *Ccl2* is significantly increased in the ipsilateral cortex in vehicle- and 10 mg/kg decernotinib-treated groups (***P < 0.01). **(H)** *Cxcl10* is significantly increased in the ipsilateral cortex in both groups (***P < 0.01; **P < 0.01). **(I)** *Cxcl1* is increased in the ipsilateral cortex in vehicle- and 10 mg/kg decernotinib-treated groups (***P < 0.01). **(J)** *IL-1*β is increased in the ipsilateral cortex in vehicle- and 10 mg/kg decernotinib-treated groups (***P < 0.01). **(K)** *IL-6* is increased in the ipsilateral cortex in vehicle- and 10 mg/kg decernotinib-treated groups (**P < 0.01; ***P < 0.01). **(L)** *Tnfα* is increased in the ipsilateral cortex in vehicle- and 10 mg/kg decernotinib-treated groups (***P < 0.01). **(M)** *Ptgs2* is increased in the ipsilateral cortex in vehicle- and 10 mg/kg decernotinib-treated groups (*P < 0.05; ***P < 0.01). **(N)** *Mmp-9* is increased in the ipsilateral cortex in vehicle- and 10 mg/kg decernotinib-treated groups (**P < 0.01) Statistical analyses performed with a two-way ANOVA and Bonferroni post-test; vehicle *n* = 5, 10 mg/kg decernotinib, *n* = 7.

We also performed qRT-PCR to more clearly define the role of JAK3 in the inflammatory context of ischemic stroke. Initially, we measured expression of the six ILs that use the common gamma chain associated with JAK3: *IL-2, IL-4, IL-7, IL-9, IL-15*, and *IL-21*. Compared to the contralateral cortex, *IL-21* mRNA significantly increased in the ipsilateral cortex in both treatment groups (Figure 3A). There was no significant change in ipsilateral *IL-21* levels in stroked mice receiving decernotinib compared with vehicle control. *IL-4* did not significantly change between groups (Figure 3B). IL-7 similarly showed a non-significant increase in the ipsilateral cortex of each group (Figure 3C). Levels of *IL-9, IL-15*, and *IL-2* did not significantly vary by hemisphere nor treatment group (Figures 3D–F). Because secondary injury is in large part mediated by infiltrating immune cells, we measured expression of several chemokines important for immune cell trafficking and found *Ccl2, Cxcl10*, and *Cxcl1* all dramatically increased in the ipsilateral cortex of both groups, with no effect of treatment (Figures 3G–I). To confirm the inflammatory environment initiated by our stroke model, we measured increased proinflammatory cytokines *IL-1*β, *IL-6*, and *Tnf*α in the ipsilateral cortex, and this did not significantly change with 10 mg/kg decernotinib (Figures 3J–L). Additionally, proinflammatory mediators *Ptgs2* and *Mmp-9* are increased in the ipsilateral cortex, with no difference in treatment (Figures 3M,N).

**Figure 4:**
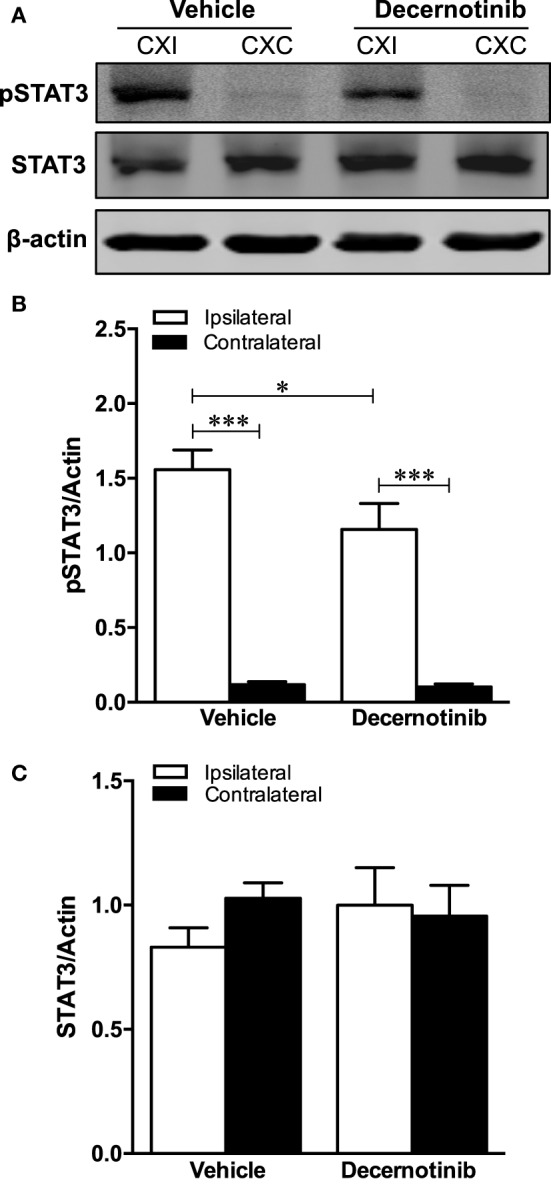
**(A)** Representative immunoblot of mouse brain tissue. **(B)** Graphical summary of phosphorylated STAT3 immunoblot data normalized to actin (*P < 0.05; **P < 0.01, vehicle *n* = 9; 10 mg/kg decernotinib, *n* = 5). Phosphorylated STAT3 is increased in the ipsilateral cortex compared to the contralateral. Phosphorylated STAT3 is decreased in the ipsilateral cortex with 10 mg/kg decernotinib compared to the vehicle-treated group. **(C)** Graphical summary of total STAT3 immunoblot data normalized to actin (vehicle, *n* = 9; 10 mg/kg decernotinib, *n* = 5). Total STAT3 does not vary between groups.

Associated with pJAK3/activated JAK3, we also determined that phosphorylated STAT3 is markedly increased in the ipsilateral cortex after ischemic stroke, and this is reduced with 10 mg/kg decernotinib (Figures 4A,B). There were no changes in total STAT3 among hemispheres or treatment groups (Figures 4A,C).

## Discussion

In this study, we investigated the effect of the specific JAK3 inhibitor, decernotinib, on acute stroke outcomes in a mouse model of focal cerebral ischemia induced by permanent ligation of the MCA. The major finding of this study is that JAK3 inhibition fails to protect the brain after stroke. We are the first to demonstrate that there is a dramatic increase in JAK3 protein levels as well as in its phosphorylation/activation state during the acute phase of ischemic stroke and that inhibiting JAK3 is ineffective at reducing stroke size compared to vehicle-treated animals using a comprehensive dose–response curve. JAK3 inhibition with decernotinib is also ineffective at reducing sensorimotor deficits after stroke.

Immunoblotting data showed that total JAK3 levels are significantly increased in the ipsilateral cerebral cortex in response to focal ischemia, which agrees with previous work identifying an increase in JAK3 mRNA after transient MCAO in rats compared to sham-operated control (4). Furthermore, we found positive JAK3 immunolabeling on neurons, microglia/macrophages, and endothelial cells in the ischemic mouse brain. These data are consistent with previous reports identifying JAK3 in neurons (19), endothelial cells (20), and cells of hematopoietic origin such as macrophages (21). Although JAK3 has been found in immunoblots from cultured astrocytes (22), we did not find JAK3 immunostaining on GFAP-positive astrocytes after stroke.

Inhibiting JAK3 appears to be a double-edged sword in the acute phase of ischemic stroke; although it has the ability of reducing *IL-17A* mRNA transcription by γδT cells and IL-21, which have been shown to be detrimental in stroke (8, 23), it is also blocking potential IL-4 signaling in neurons, which is neuroprotective in the ischemic brain (24).

To further confirm that JAK3 inhibition is not neuroprotective in our model of ischemic stroke, we performed qRT-PCR to measure potential changes in ILs that use the common gamma chain associated with JAK3 and found no significant difference in the ipsilateral cortex between vehicle- and 10 mg/kg decernotinib-treated groups. In the same vein, JAK3 inhibition with 10 mg/kg decernotinib did not affect stroke-induced upregulation of *Ccl2, Cxcl10*, and *Cxcl1*, chemokines important for immune cell trafficking, nor proinflammatory mediators *IL-1*β, *IL-6, Tnf*α, *Ptgs2*, and *Mmp-9* that exacerbate stroke damage.

We are the first to demonstrate that pMCAO with our model increases *IL-21* expression in the ipsilateral cortex, and this increase may at least partially account for the increased JAK3/STAT3 phosphorylation in this region (25, 26). Elevated *IL-21* is similarly found in models of transient ischemia 24 h later (23). Although the actions of *IL-21* on infiltrating T-cells result in an overall deleterious outcome in stroke, *IL-21* is important for angiogenesis and cell survival in endothelial cells subjected to hypoxia/serum starvation by activating STAT3 which increases antiapoptotic BCL-2 (27).

In addition to increased *IL-21* expression after stroke, we also found increased phosphorylated STAT3 which was decreased with decernotinib treatment. This is in line with a previous report showing that tofacitinib, a JAK1/JAK3 inhibitor, abolishes IL-21 induced STAT3 phosphorylation in HUVECs subjected to hypoxia/serum starvation (27). Furthermore, increased phosphorylated STAT3 24 h after MCAO has been reported before in other rodent models of ischemic stroke (28–30).

Our results differ from another report in a transient intraluminal MCAO model claiming that tofacitinib reduced neurological deficits and preliminary data in which infarct size was not measured (8). This is in part due to that fact that tofacitinib inhibits JAK1/JAK3, but decernotinib selectively inhibits JAK3. Additionally, our model of stroke induces damage limited to the cerebral cortex, and results from permanent rather than transient MCAO which is associated with reperfusion injury. We studied the effects of decernotinib on acute stroke outcomes in permanent ischemic stroke since a large percentage of human ischemic stroke cases fail to qualify for reperfusion therapy, and brain damage results from permanent occlusion of major cerebral vessels (31–33). One limitation of this investigation is that we only analyzed the effect of decernotinib administration during the acute phase of stroke (24–48 h) rather than longer term (>7 days). Although in this model of permanent stroke, the infarct does not appreciably spread between 2 and 14 days postischemia (unpublished data from our laboratory), there would be significant infiltration of T-cells during this time period (34). Previous stroke studies have shown that blocking T-cell infiltration is protective in stroke (7), and JAK3 KO mice have non-functional T-cells, so if JAK3 inhibition was neuroprotective, it would most likely be due to its effect on T-cells.

Despite our findings of a lack of protection by JAK3 inhibition in a mouse pMCAO model, JAK3 blockade remains a potential target to minimize neuroinflammation in the ischemic brain. Future studies should investigate the effects of decernotinib in stroke models associated with reperfusion as well as the impact of JAK3 inhibition on long-term outcomes in embolic stroke models with and without thrombolysis with recombinant tissue plasminogen activator.

In conclusion, the present study shows that JAK3 is expressed on neurons, microglia/macrophages, and endothelial cells in the mouse brain after ischemic stroke. Total and pJAK3/activated JAK3 and phosphorylated STAT3 levels dramatically increase after ischemic stroke in addition to upregulated *IL-21* mRNA. We show effective suppression of JAK3 and STAT3 phosphorylation in the mouse brain by decernotinib treatment. However, globally inhibiting JAK3 with various doses of decernotinib is not neuroprotective in a mouse model of focal ischemic stroke as evidenced by qRT-PCR of proinflammatory mediators, behavioral tests, and infarct calculation.

## Ethics Statement

All animal procedures were performed in accordance with approved guidelines of the National Institutes of Health for the Care and Use of Laboratory Animals, the ARRIVE guidelines (https://www.nc3rs.org.uk/arrive-guidelines), and the guidelines approved by the Institutional Animal Care and Use Committee (IACUC) at the University of Florida (protocol #201607934).

## Author Contributions

KD, SP, CY, DS, and EC-J performed experimental procedures. KD and EC-J designed research and planned all the experiments. KD, CY, and EC-J analyzed the data and prepared the figures. KD and EC-J wrote the article. EC-J conceived and led the project. All the authors read and approved the final version of the paper.

## Conflict of Interest Statement

The authors declare that the research was conducted in the absence of any commercial or financial relationships that could be construed as a potential conflict of interest.

## Abbreviations

JAK3, Janus kinase 3; MCA, middle cerebral artery; IL, Interleukins; TTC, 2,3,5-triphenyl-tetrazolium chloride; TBS, Tris-buffered saline; PBS, phosphate-buffered saline; pMCAO, permanent middle cerebral artery occlusion.
